# The Impact of Interaction between Body Posture and Movement Pattern Quality on Injuries in Amateur Athletes

**DOI:** 10.3390/jcm13051456

**Published:** 2024-03-02

**Authors:** Dawid Koźlenia, Katarzyna Kochan-Jacheć

**Affiliations:** Faculty of Physical Education and Sport, Wroclaw University of Health and Sport Sciences, I.J. Paderewskiego 35, 51-612 Wroclaw, Poland; katarzyna.kochan-jachec@awf.wroc.pl

**Keywords:** injury, amateur athletes, movement pattern quality, body posture, asymmetry

## Abstract

**Background**: this study aimed to examine the impact of interaction between body posture and the quality of movement patterns on injury frequencies in amateur athletes. **Methods**: The study sample consisted of 89 young amateur athletes. Movement pattern quality was assessed by the Functional Movement Screen (FMS), test and body posture in the frontal plane was assessed by the moire method for the parameters Shoulder Slope Angle, Lower Scapula Protrusion Difference, and Pelvic Tilt Angle. Injury data were collected through completion of the Injury History Questionnaire for the past 12 months. **Results**: Using cluster analysis, participants were allocated into a either category with good (BPg) body posture or poor (BPp), and using FMS cutoff points (14), either a category of good movement pattern quality (MPg) or poor (MPp). Two-way ANOVA was performed, and the Bonferroni post-hoc test revealed a reduction in injuries among participants from the MPg-BPg group compared to the other three groups (*p* < 0.05). However, no interaction between factors was revealed. No statistically significant differences were observed among the remaining three groups in the case of injury prevalence (*p* > 0.05). **Conclusions**: A combination of proper body posture and high-quality movement patterns is associated with a lower frequency of injuries, without direct interaction between chosen factors, which suggests that they impact injury risk independently. Practicing suitable BP and ensuring high-quality MPs should be regarded as a strategy in injury prevention.

## 1. Introduction

Professional or amateur sports or recreational activity is inherently associated with a risk of musculoskeletal injury [1,2]. The occurrence of injuries is prevalent among individuals engaged in physical activity regardless of age and sex [3,4]. In sports, injuries are the most common reasons for abandoning or discontinuing a sports career [5]. In amateur or recreational sports, injury can lead to discouraging physical exertion, or fearing its initiation. They occur in both experienced and amateur individuals, with the greatest risk affecting the youth and young adults [6,7].

It has been demonstrated that specific types of injuries are associated with different physical activity type and sport disciplines, and most of them temporarily exclude athletes from training and competitive activities, leading to a reduction in their physical potential [8]. However less research has been conducted on injury rates among amateur athletes for whom sports are not a source of livelihood but still an important part of their lifestyle [9].

Identifying factors associated with injuries is crucial for the implementation of effective prevention methods. Analyses are conducted concerning extrinsic (overuse effort, too high loads, weather, or low-quality equipment) and intrinsic (sex or age-specific conditions, low muscle strength, or overweight) risk factors for injuries [10]. Musculoskeletal injuries typically arise when there is an overload on the musculoskeletal structures that surpasses their capacity for regeneration or adaptation [11]. Numerous factors can influence musculoskeletal load, thereby increasing the risk of injury. The vast majority of injuries result from a combination of these factors, making it challenging to pinpoint a single specific mechanism leading to injury. Intrinsic risk factors can be described as are non-modifiable (such as age or previous injury), while others are modifiable such as strength, range of motion, and motor control [1]. These modifiable intrinsic risk factors are reflected in movement pattern quality and body posture [12,13,14]. While focusing on specific areas of injury risk, these factors provide valuable insights into the state and function of the musculoskeletal system. Therefore, assessing these indicated factors and implementing appropriate preventive measures can effectively address underlying issues and reduce the risk of injury [15,16]

Numerous studies have analyzed the relationship between injuries and Functional Movement Screen (FMS) test results, indicating movement dysfunctions [12,13]. High-quality movement patterns guarantee the safety of movement and motor efficiency, resulting from the correct construction and function of the body. Movement patterns allow for inferences about functional asymmetry. However, this asymmetry could be related to the static posture of the body [17]. Theoretically, correct body posture, both in terms of frontal plane symmetry and the absence of spinal defects, ensures that muscles and joints function properly, enabling the display of the correct movement patterns [17]. Body posture is therefore one of the factors allowing for functional stabilization, which can be crucial in physical activity. However, the relationship between body posture and movement patterns is still unexplored, and existing results are inconclusive [17,18].

Current research unequivocally indicates that training loads can lead to structural changes in the musculoskeletal system specific to a particular sports discipline [16,19,20,21]. Even amateur athletes exhibit changes in the formation of anterior–posterior spinal curves [22,23,24,25]. Findings from other studies indicate pelvic asymmetry in female soccer players [23], and asymmetry in shoulder blades, waist triangles, shoulder lines, and spines in young track and field athletes [24,26] and tennis players [27]. The impact of hyperkyphosis on lowering the shoulder and indirectly on subacromial impingement syndrome in football players has also been identified [28]. Another study of athletes in the same sport showed that kyphotic and lordotic posture may negatively affect neuromuscular explosiveness performance, while lateral spine curvature reduces lower limb flexibility [29].

To the best of our knowledge, there are few studies analyzing the impact of postural dysfunctions on injury frequency [30,31]. Snodgrass et al. demonstrated a relationship between lower limb injuries and kyphosis [32]. Retrospective studies involving over 20,000 individuals with abnormal spinal shapes suggest that they are significantly more susceptible to injuries than individuals with correct body posture [31]. Results from several studies confirm that postural dysfunctions may contribute to functional disorders manifesting at various levels of the biokinetic chain, potentially hindering the execution of proper movement patterns. Such relationships were observed in hockey players, showing a correlation between the magnitude of anterior–posterior spinal curves and FMS results [17], as well as in military personnel regarding postural irregularities in the sagittal and frontal planes [33]. However, studies conducted on 8–11-year-old children [18] and young women with hyperlordosis [34] did not confirm such relationships, which indicated the need for further exploration.

There is a lack of research comprehensively addressing the quality of movement patterns and body posture as injury risk factors. As was demonstrated above, there are associations between FMS test results and the occurrence of injuries [15], as well as between FMS results and body posture [17]. Perhaps these factors could interact with each other, and better movement patterns as well body posture may act additively as protective factors against injuries. However, to date, there is a lack of studies exploring the interaction between these factors in the context of injury risk. Obtaining comprehensive knowledge about injury risk determinants may contribute to identifying ‘weak links’ in the biokinetic chain, while allowing for the integration of individually tailored preventive exercises into training. Therefore, the aim of this study was to examine the impact of interaction between body posture and the quality of movement patterns on injury frequencies in amateur athletes. Specifically, we aimed to (1) compare the potential effects of movement patterns and body posture on the risk of injury and (2) assess the interaction effect between body posture and the quality of movement patterns and injury frequencies. Based on provided observations, we hypothesized that body posture significantly contributes to explaining the risk of injuries, especially when considered in conjunction with the quality of movement patterns. The chosen factors could interact and therefore effectively identify increased injury occurrence.

## 2. Materials and Methods

### 2.1. Participants

This study involved a sample of 89 active young adults aged 19–23 years with a minimum of 4 years of sports experience and engaged in training at least three times per week. Among these, men (*n* = 41) exhibited a body height of 1.82 ± 0.05 cm, body weight of 79.42 ± 8.2 kg, and BMI 23.90 ± 2.00 kg/m^2^, and 36.59% of men had been injured in the past 12 months, while women (*n* = 48) displayed a body height of 1.67 ± 0.06 cm, a body weight of 58.96 ± 7.75 kg, and BMI 20.91 ± 2.13 kg/m^2^, where 41.06% of women had been injured. Participants were involved in regular participation in sports such as soccer, futsal, handball, basketball, and rugby. To maintain test integrity, individuals who were currently injured (*n* = 9) during study commencement were excluded. All subjects were obligated to provide written consent and received comprehensive information about the study’s objectives, methodology, and participation requirements. Participants were allowed to withdraw from the research at any point without the need for explanation.

### 2.2. Handling and Imputation of Missing Data

There were no missing data in the anthropometrical measurements, FMS assessment, and injury questionnaire, but there were missing data in body posture measurements (*n* = 7). It was necessary to keep the same number of participants, and the statistical methods used in the study, cluster analysis and analysis of variance, require there to be no missing data. All measurements were preprocessed by applying multiple imputations. The propensity for a data point to be missing was completely random, also known as missing completely at random [35]. There was no relationship between whether a data point was missing and any other values in the data set. Imputation was conducted in R language using RStudio software v. 2023.06.0+421 (RStudio Team (2023)) [36].

### 2.3. Measurements

Body measurements. Body height and weight were measured with a SECA 764 device (SECA GMBH & CO., Hamburg, Germany), and based on the received parameters, the body mass index (BMI) was calculated: BMI = body weight [kg]/body height [m^2^].

Injury. The study gathered injury data from participants using the Injury History Questionnaire (IHQ). This is a reliable tool used in research [37]. The survey was administered under supervision, with the researcher available to address any uncertainties or doubts that participants may have had while completing the questionnaire. Injury was defined as the occurrence of complaints during physical activity resulting in pain and discomfort in the locomotor system and causing temporary limitations or a complete inability to continue physical activity [38]. The questions required answers about a number of injury experiences in specific body areas (head and neck, trunk, and left and right upper and lower limbs). The total number of injuries were collected in a specified time frame (within the past 12 months). The evaluation of IHQ reliability occurred seven days after participants completed the IHQ. A random sample of 56 individuals underwent a repeated survey. IHQ reliability was assessed using the alpha Cronbach coefficient, which indicated high reliability at a level of 0.836 [35].

Movement pattern quality. The FMS test was employed to evaluate movement pattern quality, involving seven tasks: deep squat (DS), hurdle step (HS), in-line lunge (IN-L), shoulder mobility (SM), active straight leg raise (ASLR), trunk stability pushup (TSPU), and rotary stability (RS) using original FMS kit (Perform Better, Cranston, RI, USA). The reliability of the test was confirmed [39]. The FMS test assesses the functional state of the locomotor system in terms of motor control, mobility, and stability. Each motor task was rated on a scale of zero to three, following clear guidelines outlined for each test [12,13]. Subjects received three points for performing the movement correctly, two points for visible compensation, and one point if the subject could not perform the movement. The FMS evaluation incorporated three supplementary tasks beyond the primary assessment to detect any signs of discomfort. Each of these tasks was executed thrice. In instances of unilateral trials, the lower score was factored into the final assessment. Consequently, the highest achievable score amounted to 21 points. Research indicates a notable escalation in injury risk when scores fall at or below 14 points [15].

Body posture. The torso frontal plane was measured using the projection moire phenomenon by a Mora 4G-HD device (CQ Elektronik System, Czernica, Poland). The method and device have been confirmed to be reliable and standardized in the procedure described by Mrozowiak [40], which was introduced. The subject was positioned with their back to the front of the camera 2.6 m away. The subject was instructed to maintain their habitual position, keeping eyes and ears in line horizontally, arms relaxed at the side of the body, and feet parallel to the platform. The participant’s picture was captured by a dedicated optical system and presented on the computer screen using the lens. The result was a picture in the form of contour lines with the so-called striae of moire. The distorted lines were recorded in the computer memory and processed by a numerical algorithm into a contour map of the surface. The parameters were calculated, and absolute values were considered in terms of distance from median plane of the body and were analyzed comprehensively.

Shoulder Slope Angle (SSA)—the acromion height difference between the median plane of the trunk was calculated. The difference between the right and left side was calculated. Values further from zero indicate greater asymmetry of the upper limb position.

Lower Scapula Protrusion Difference (LSPD)—lower scapula angles were marked and the distance from the median plane of the trunk positioned on the spine was calculated. Then, the difference between the right and left side was calculated. Values further from zero indicate greater asymmetry of the upper limb position.

Pelvic Tilt Angle (PTA)—this parameter determines the inclination of the line connecting points between the superior posterior iliac spine (SPIS) relative to the horizontal (angle is horizontal). It takes values in degrees within the range (−180 to +180). Therefore, if the right SPIS is ‘higher’ than the left SPIS, the angles are within (0–180), otherwise they are within (−180–0). The value given in millimeters represents the height difference between the positions of the points. Values further from zero indicate greater asymmetry of pelvic position.

### 2.4. Statistical Analyses

The Shapiro–Wilk test for normality of the distribution, as well as Levene’s test for homogeneity of variances were conducted. All quantitative data were expressed as mean ± SD and 95%CI, while qualitative data were presented as numbers and percentages. Hierarchical cluster analysis was conducted to classify participants as those with good and poor body posture. Three variables, SSA, LSPD, and PTA, identified previously as predictors of injury were used in the clustering algorithm. Ward’s method and Euclidean distances were used. The cluster analysis method effectively distinguished participants with poor and good body posture parameters. Based on the FMS overall results and the cutoff point, participants with a lower (*n* = 43) and higher risk of injury (*n* = 46) were identified (cutoff = 14) [l5]. The dependent variable was the number of injuries during the year before examination. Because of large skewness, the transformation of the variable was conducted with one of the power transformation methods using the Yeo–Johnson algorithm [41]. Two-way ANOVA was used to conduct the analysis. The main effects (FMS and BP) and their interaction effect were studied. Detailed comparisons were conducted with post-hoc tests with Bonferroni correction applied. The significance level was assumed in all statistical tests to be α = 0.05. Statistica v13.0 (Statsoft Polska, Cracow, Poland) was used for the analyses.

## 3. Results

Table 1 presents descriptive statistics of analyzed variables in body posture and movement pattern assessment.

**Figure 1:**
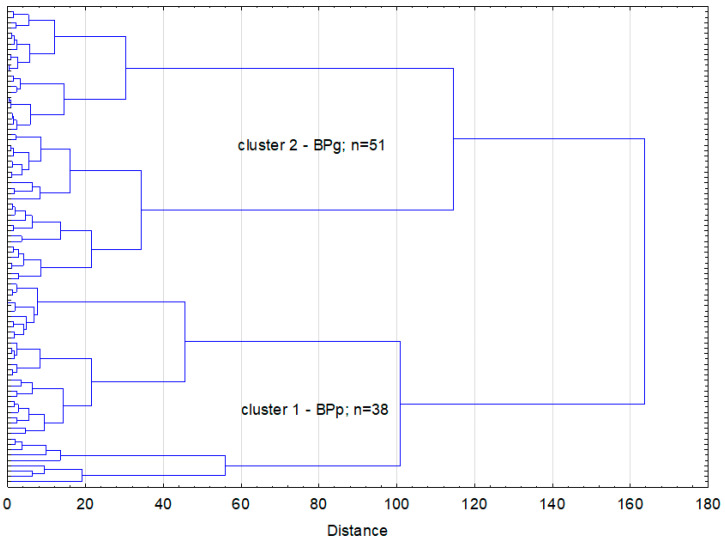
Cluster analysis based on body posture parameters in frontal plane.

The cluster analysis based on chosen body posture parameters revealed two clusters, with one including 38 individuals with poor BP, whereas the second cluster contained 51 people classified as having good BP (Figure 1).

**Figure 2:**
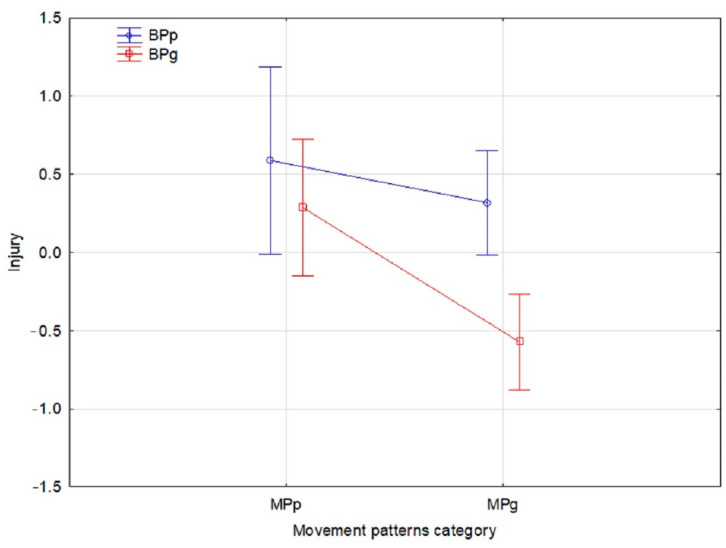
Mean values and 95%CI in FMSg-BPg, FMSp-BPg, FMSg-BPp, and FMSp-BPp.

Then, we considered movement pattern quality, and further division of the participants was performed on the four groups using two-way ANOVA, which showed a significant main effect of the FMS ((F)_ = 6.69, *p* = 0.013), as well as a significant main effect of BP ((F) = 7.46, *p* = 0.008). Both factors had a medium effect on the number of injuries, which was confirmed by eta squared partial (η^2^_p_ = 0.073, η^2^_p_ = 0.081, respectively). However, there was no significant interaction effect ((F) = 1.82, *p* = 0.180, η^2^_p_ = 0.021, small effect size). Mean values and 95%CI of the four groups are presented in Figure 2. FMSg-BPg *n* = 25; FMSp-BPg *n* = 26; FMSg-BPp *n* = 21; FMSp-BPp *n* = 23.

Detailed comparisons between all four groups, with a post-hoc test using Bonferroni correction, showed significantly less injuries in participants with good movement quality patterns and good body posture (FMSg-BPg) compared to the other three groups: FMSp-BPp (*p* = 0.006), FMSp-BPg (*p* = 0.001), and FMSg-BPp (*p* = 0.001). However, there were no statistically significant differences between the remaining three groups (*p* > 0.05).

## 4. Discussion

Injury prevention is one of the most significant challenges for popularizing physical activity. Accurate identification of factors contributing to injuries is vital in selecting the appropriate method of injury prevention. It is also important to take a multifactorial approach that considers many factors that may contribute to injuries. Therefore, this study showed an injury connection with movement quality combined with body posture. The complex view of injury occurrence considers that these factors could indicate an effective method of injury prevention. Our results revealed that a combination of high-quality movement patterns and good body posture constitute a relevant factor in reducing injury frequency, despite both factors having a weak interaction.

Injury risk among amateur athletes is a significant issue [2,4]. Many studies have confirmed a high prevalence of injury among various groups of physically active individuals [6,7]. Prieto-Gonzalez [42] indicated that increased injury risk occurs due to high training load and intensity, age, skipping warm-ups, and inadequate sports facilities. To reduce injuries in youth sports, preventive programs should address these factors as well identifying other possible ones and the interaction between them, as injuries are multifactor phenomena [1].

The risk of injury is inherent in various physical activities, irrespective of their type or intensity [43,44]. Accurate prediction of injuries enables individuals to proactively adopt preventive measures [45]. Physical activity has an influence on body posture, especially when some specific demands associated with movement and behaviors occur such as in combat sports [21]. Male and female volleyball players at the university level exhibit significant asymmetry in rotation strength between their dominant and non-dominant arms [46]. These could be considered intrinsic injury risk factors due to overuse injury being associated with higher efforts in one body area, whereas other areas could be weak. In terms of movement patterns, Garrison et al. [47] demonstrated the predictive accuracy of the Functional Movement Screen (FMS) test, revealing a nearly 6-fold higher injury risk for those with lower-quality movement patterns and a 15-fold increase for individuals with previous injuries. Similar trends were observed in studies of rugby players [48] and athletes [49]. The study by Koźlenia and Domaradzki [37], with a 73% prediction accuracy, aligns with these findings. However, some studies question the accuracy of injury prediction using the FMS test [50]. Holistic approaches encompassing various factors are essential. Considering the common influence of movement patterns and body posture on injuries is crucial, as demonstrated by our finding that participants with high-quality movement patterns and proper body posture are less likely to be injured. Recognizing the interplay between movement patterns and body posture in particular could enhance injury prediction. However, attempts to predict injuries based on these factors individually or simultaneously have yielded mixed results.

Many sports, characterized by specific body postures and enforced patterns, expose practitioners to training adaptations that can impact body posture, particularly altering the spinal curvature [17]. While noticeable developmental changes in posture may not occur in mature individuals, scientific studies suggest that years of practicing such sports can lead to postural changes. The resulting abnormal body posture and associated muscle tension often manifest as pain, commonly reported in areas like the head, shoulder girdle, and spine. Moreover, these issues can contribute to degenerative spine diseases and even heart arrhythmias [51]. Identifying postural abnormalities and their causes becomes crucial for understanding the etiology of pain and joint mobility restriction injuries, and developing corrective exercise strategies [52]. Studies on the use of specific sports to counteract postural disorders indicate that even in adult athletes, positive and lasting changes can be achieved through body posture re-education. For instance, a study by Kaplan [53] demonstrated positive effects on asymmetrical posture after 12 weeks of football training among basketball players. Małek et al. [54] found that triathlon training contributes to maintaining normal postural muscle length, increased spine and chest mobility, and improved overall body posture. Notably, individuals who had previously trained in sports involving asymmetrical movements and later took up triathlon training showed improved deep stabilizers, highlighting the potential benefits of varied physical activities on postural health.

There is a need to remember that injury is multifactor phenomenon, and therefore, there is a need to consider other factors [55]. In our study, we analyzed also body posture parameters, which remain relatively unexplored in the case of injury risk. A previous study considers body posture as a possible factor of injury risk [56]. However, not many studies have provided such analysis, which makes it difficult to discuss our study with other results. Research suggests a connection between injuries and forward trunk lean in taekwondo athletes, as well as kyphosis [30,32]. Individuals with an abnormal spinal shape are more prone to injuries [31]. Asymmetry in skeletal muscle mass increases the risk of injury in physically active individuals [57]. Postural dysfunctions may contribute to functional impairments and hinder the execution of proper movement patterns [33]. Also, few studies have provided evidence for an association between movement patterns and body posture. The study by Mitchell et al. [18] conducted on children showed that movement patterns are associated with body posture. Also, Uzer et al. [58] showed that postural irregularities in wrestlers, particularly those affecting the vertebral column, have the potential to disturb the biomechanical alignment of both upper and lower extremities. This disruption may lead to joints being unable to execute good quality movements at optimal angles, consequently elevating the likelihood of injury among wrestlers. However, in our study, we did not show interactions between these factors, which suggests their independent influence on injury risk; however, there was no difference between groups, with one factor being good and the second one being poor, which showed a comparable meaning of this factor in injury risk, whereas both factors scoring on a good level provided lower injury risk.

Our study was limited to retrospective data, and therefore, future studies should also consider a prospective approach. Extending the study sample to include a wider range of ages as well into specific subpopulations of professional athletes, additionally in terms of potential sex differences, would indicate the study’s finding’s utility in more universal settings. Caution is warranted considering the influence of other intrinsic and extrinsic factors on injury risk. Future studies should broaden their analysis to include additional potential factors that may impact injury mechanisms. On the other hand, to date, there is lack of data considering movement pattern quality assessment combined with body posture as injury risk factors, despite these factors being treated as a base expression of the human body’s state and function. We also recommend considering other parameters describing body posture to verify its usefulness in injury risk evaluation and validate the cutoff point showing increased injury risk [10,15]. Our approach expands on current knowledge considering intrinsic injury risk factors and indicates further avenues of research. Our findings encourage the initiation of future studies aimed at addressing body posture abnormalities and low-quality movement patterns through targeted training programs and verifying the effectiveness of these approaches in prospective terms.

## 5. Conclusions

Our results reveal a unique observation due to the lack of previous studies that consider movement pattern quality and body posture in one model of assessment in the injury risk context. Despite high-quality movement patterns and good body posture being associated with decreased injury occurrence, surprisingly, the factors affected injury independently due to a lack of interaction between them. Therefore, our hypothesis was partially confirmed. Maintaining proper body posture and consistently executing high-quality movement patterns have been related to a reduced incidence of injuries. Consequently, prioritizing the development of appropriate body posture and promoting consistent adherence to high-quality movement patterns should be recognized as a fundamental strategy in injury prevention. Practitioners should introduce methods associated with improving movement pattern quality as well providing correcting strategies for maintaining appropriate body posture. Due to the lack of interaction between movement patterns and body posture, independent methods of development should be provided.

## Figures and Tables

**Table 1:** Descriptive statistics of body posture parameters and FMS score.

Variable	Men	Women
	Mean ± SD (95%CI)	Mean ± SD (95%CI)
FMS [points]	14.22 ± 2.95 (13.29–15.15)	14.77 ± 3.02 (13.89–15.65)
SSA[mm]	6.19 ± 7.14 (3.93–8.44)	4.55 ± 10.66 (1.19–7.92)
LSPD [mm]	6.19 ± 7.14 (3.93–8.44)	4.55 ± 10.66 (1.19–7.92)
PTA[mm]	−0.7 ± 2.39 (−1.46–0.05)	0.56 ± 2.95(−0.37–1.49)

Abbreviation: FMS—Functional Movement Screen; SSA—Shoulder Slope Angle; LSPD—Lower Scapula Protrusion Difference; PTA—Pelvic Tilt Angle.

## Data Availability

The data presented in this study are available upon request from the corresponding author.
